# Biatrial Approach Provides Better Outcomes in the Surgical Treatment of Cardiac Myxomas

**DOI:** 10.5935/1678-9741.20160066

**Published:** 2016

**Authors:** Ahmet Yüksel, Davit Saba, Yusuf Velioğlu, Serdar Ener, Hayati Özkan

**Affiliations:** 1 Uludag University Faculty of Medicine, Bursa, Turkey.; 2 Abant Izzet Baysal University Faculty of Medicine, Bolu, Turkey.; 3 Acıbadem Bursa Hospital, Bursa, Turkey.

**Keywords:** Myxoma, Cardiac Surgical Procedures, Methods, Death, Sudden, Cardiac

## Abstract

**Objective:**

We aimed to present clinical features, surgical approaches, importance of surgical technique and long-term outcomes of our patients with cardiac myxoma who underwent surgery.

**Methods:**

We retrospectively collected data of patients with cardiac myxoma who underwent surgical resection between February 1990 and November 2014. Biatrial approach is the preferred surgical method in a large proportion of patients that are operated due to left atrial myxoma because it provides wider exposure than the uniatrial approach. To prevent recurrence during surgical resection, a large excision is made so as to include at least 5 mm of normal area from clean tissue around the tumor. Moreover, special attention is paid to the excision that is made as a whole, without digesting the fragment of tumor with gentle dissections.

**Results:**

Forty-three patients (20 males, mean age of 51.7±8.8 years) were included. Most common symptom was dyspnea (48.8%). Tumor was located in the left atrium in 37 (86%) patients. Resections were achieved via biatrial approach in 34 patients, uniatrial approach in 8 patients, and right atriotomy with right ventriculotomy in 1 patient. One patient died due to low cardiac output syndrome in the early postoperative period. Mean follow-up time was 102.3±66.5 months. Actuarial survival rates were 95%, 92% and 78% at five, 10 and 15 years, respectively. Recurrence was observed in none of the patients during follow-up.

**Conclusion:**

Although myxomas are benign tumors, due to embolic complications and obstructive signs, they should be treated surgically as soon as possible after diagnosis. To prevent recurrence, especially in cardiac myxomas which are located in left atrium, preferred biatrial approach is suggested for wide resection of the tumor and to avoid residual tumor.

**Table:** 

Abbreviations, acronyms & symbols	
AF	= Atrial fibrillation
IABP	= Intraaortic balloon pump
ICU	= Intensive care unit
NYHA	= New York Heart Association
TEE	= Transesophageal echocardiography
TTE	= Transthoracic echocardiography

## INTRODUCTION

Primary cardiac tumors are rare neoplasms with an autopsy incidence between 0.001% and 0.3%^[1-3]^. Approximately 75-80% of primary cardiac tumors are benign, and of those, more than half are myxomas^[1-6]^. Myxomas occur in all age groups, but are more likely to present between the third and sixth decades of life. Myxomas are predominantly present in females and, in the majority of patients, they originate from the region of fossa ovalis of interatrial septum in the left atrium^[7-10]^.

Although myxomas are histologically benign, this type of tumor carries the risk of systemic embolization with subsequent cerebral or peripheral infarctions, intracardiac obstructions, syncope, and sudden death^[11,12]^. Therefore, surgical treatment for cardiac myxomas should be performed as soon as possible after diagnosis. The basic principles of surgical treatment for cardiac myxomas include complete wide excision of tumor and avoidance of residual tumor. However, the most appropriate surgical approach especifically for left atrial myxomas is controversial.

We aimed to present clinical features, surgical approaches, importance of surgical technique and long-term outcomes of patients with cardiac myxoma who underwent surgery in this study.

## METHODS

From February 1990 to November 2014, a total of 65 patients were operated due to primary cardiac tumors in our institution. Of those, 43 patients had undergone surgery for cardiac myxoma. In this study, the patients who had undergone surgical resection for cardiac myxoma were included in the study and their records were reviewed retrospectively. The study protocol was approved by the Ethics Committee of the Faculty of Medicine, Uludag University. As this was a retrospective study involving routine cardiac surgical procedures, informed patient consent was not required; however, the approval for screening of patient files was obtained from the Ethics Committee.

### Preoperative Evaluation and Diagnosis

In the presence of clinical suspicion of a diagnosis of cardiac tumor, diagnostic studies were initiated. Preoperative diagnosis was established in all patients by two-dimensional transthoracic echocardiography (TTE). Transesophageal echocardiography (TEE) was performed in patients whose diagnosis was doubtful on TTE. Furthermore, TEE was routinely performed intraoperatively to inspect all four cardiac chambers. In some cases, additional computed tomographic scanning and magnetic resonance imaging were performed to obtain the diagnosis. Coronary angiography was performed if the patient had a history of chest pain or was older than 40 years old. Preoperative routine laboratory investigations in myxoma patients consisted of full blood count and blood biochemistry, including erythrocyte sedimentation rate and C-reactive protein.

### Surgical Technique

An operation was performed in all cases as soon as possible after diagnosis of cardiac myxomas was established. Surgical resection was performed through a median sternotomy incision in all cases. Cardiopulmonary bypass was conducted with aorticbicaval cannulation, mild or moderate systemic hypothermia, aortic cross-clamping, and antegrade cardioplegic cardiac arrest. Manipulation of the heart before the aortic cross-clamping was minimized in deference to the known friability and embolic tendency of myxomas. If the tumor is in the typical location in the left atrium, left ventricular venting through the left superior pulmonary vein is not performed, to avoid dislodging tumor material. There was no requirement for deep hypothermic circulatory arrest in any of the cases with right atrial myxoma.

The surgical approach was selected according to the localization and size of myxoma, the preference and experience of the surgeon, the presence of concomitant cardiac diseases, and the genetic nature of the myxoma. A standard right atriotomy approach was performed for all of the right atrial myxoma cases. A right atriotomy with right ventriculotomy was performed for only one right atrial plus right ventricular myxoma case.

The surgical approaches for left atrial myxomas were divided into two groups, according to the number of opened atrial chambers: uniatrial and biatrial. The uniatrial approach for left atrial myxomas was classical left atriotomy. The biatrial approach group included patients who underwent right atrial transseptal approach, biatriotomy, and superior transseptal approach. With the uniatrial approach, preoperative and intraoperative echographic confirmation of the absence of contralateral myxoma was obtained. With the biatrial approach, both atria and ventricles were carefully inspected for tumor fragments or other myxomas. The uniatrial approach was preferred in a few patients with left atrial myxoma, who had particularly small tumors. The biatrial approach was the preferred form of surgery in a large proportion of the patients that were operated on due to left atrial myxoma because it provides wider exposure than the uniatrial approach.

The main objective of resection was complete excision of the tumor together with removal of the attachment base in order to prevent recurrence, with a full-thickness resection in all of the cases. However, in few cases, subendocardial intramural resection was utilized when a full-thickness resection would have led to disruption of structural or functional integrity.

The right atrial transseptal approach involved an oblique right atriotomy to approach the interatrial septum and incise the fossa ovalis initially at the limbus to expose the myxoma. The exposure through this incision in the interatrial septum was facilitated further by using small retractors and, if required, by applying gentle pressure to the lateral wall of the left atrium. The biatriotomy approach involved a longitudinal incision on the left atrial wall, posterior to the interatrial groove, and a counter incision on the right atrial wall. The right atrial incision is standard, approximately 1 cm parallel to the atrioventricular groove. This exposure usually results in adequate immediate visualization of the tumor. The superior transseptal approach involved an incision on right atrial mid-lateral wall, extending anteriorly through the midline of the right atrial appendage, then posteriorly down the back of the appendage to the superior end of the interatrial septum. The fossa ovalis was visualized and the interatrial septum was incised. The incision was extended cephalad to join the previous incision at the superior end of the septum. The left atrial dome was entered at the junction of the two previous incisions. The entire tumor mass, the attachment of the pedicle of the myxoma, as well as the mitral valve annulus were visualized clearly through these surgical approaches. Then, the tumor and its attachment was widely removed, taking care not to injure the mitral annulus, the area of conduction tissue, and the tricuspid annulus. In some cases, when the tumor was larger, gentle pressure to the right ventricular outflow tract area was applied to aid in exposing the stalk of the tumor. In order to avoid recurrence, a wide resection was carried out so as to include at least 5 mm normal area from clean tissue around the tumor. In addition, especial attention was paid to the excision that was made as a whole without digesting the tumor fragments, with minimal manipulation and gentle dissections. All cardiac chambers were meticulously inspected in order to avoid residual tumor fragments. The chambers were copiously irrigated with cold saline solution to eliminate any loose fragments that may have dislodged during the removal of the tumor. The surgically created atrial septal defects were repaired by direct suture or using a pericardial or Dacron patch (C.R. Bard Inc., Murray Hill, NJ, USA). De-airing and the remainder of the procedure were completed in the standard fashion.

### Histopathological Examination

All resected tumors were sent to histopathological examination, and the diagnosis of myxoma was confirmed in all cases. Histopathological examination revealed proliferations of capillaries, blood extravasations, and disseminated fibrin deposition. These findings were consistent with the diagnosis of a myxoma.

### Follow-Up Data

All patients were followed up on an outpatient basis at regular intervals. Clinical examination, chest radiographs, electrocardiography and TTE were performed routinely at each follow-up visit. The first visit was performed after discharge, at the latest within 3 months postoperatively; then, the other follow-up visits were performed routinely every year. Telephone interviews were required for 5 patients with follow-up visits in excess of 12 months or for those who missed the ambulatory follow-up. Three of the patients in this study were lost to followup after being discharged.

### Statistical Analysis

Statistical analysis was performed using the SPSS version 15.0 for Windows (SPSS Inc., Chicago, IL, USA) software. The data were calculated as mean ± standard deviation for continuous variables and as numbers with percentages for categorical variables. The long-term cumulative survival analysis was performed using the Kaplan-Meier method.

## RESULTS

Clinical and demographic data of patients are shown in Table 1. The study group was comprised of 23 female and 20 male patients, with a mean age of 51.7±8.8 (range: 22–76) years at the time of operation. The most common symptoms at admission were: dyspnea (48.8%), palpitation (37.2%), and chest pain (20.9%). Furthermore, the constitutional symptoms and signs of a generalized disease such as fever, fatigue and weight loss were common, being observed in 34.9% of the patients. Nine (20.9%) patients also showed symptoms of systemic embolization, either to the central or peripheral nervous system. Seven (16.3%) patients were asymptomatic. The mean duration of symptoms was 4.4±2.8 (range: 1–12) months. On physical examination, systolic murmur was audible in 13 patients and diastolic murmur in 4. Three patients had the so-called characteristic diastolic "tumor plop". Ten (23.3%) patients had a high erythrocyte sedimentation rate, 6 patients (13.9%) had a high C-reactive Protein level, and anemia was present in 4 (9.3%) patients preoperatively. None of the patients had a familial myxoma nor Carney complex syndrome.

**Table 1:** Clinical and demographic data of patients.

Localization	Left atrium	Right atrium	Right atrium and ventricle
No. of patients	37 (86%)	5 (11.6%)	1 (2.3%)
Mean age [range] (years)	52.3 [22-76]	50.2 [43-62]	37
Male/Female	16/21	03/fev	1/0
**Symptoms at admission**			
Dyspnea	19 (51.4%)	1 (20%)	1 (100%)
Palpitation	14 (37.8%)	2 (40%)	__
Chest pain	7 (18.9%)	1 (20%)	1 (100%)
Constitutional	12 (32.4%)	3 (60%)	__
History of embolization	8 (21.6%)	1 (20%)	__
**Physical examination findings**			
Systolic murmur	10 (27%)	2 (40%)	1 (100%)
Diastolic murmur	3 (8.1%)	1 (20%)	__
Tumor plop	3 (8.1%)	__	__
**New York Heart Association (NYHA) Class**			
I	5 (13.5%)	2 (40%)	__
II	30 (81.1%)	3 (60%)	__
III	__	__	1 (100%)
IV	2 (5.4%)	__	__
**Comorbidities**			
Hypertension	12 (32.4%)	2 (40%)	__
Diabetes mellitus	7 (18.9%)	__	__
Hyperlipidemia	7 (18.9%)	1 (20%)	__
Coronary artery disease	9 (24.3%)	2 (40%)	__
Chronic renal failure	2 (5.4%)	1 (20%)	__
Chronic obstructive pulmonary disease	1 (2.7%)	__	__
Chronic atrial fibrillation	3 (8.1%)	__	__

The majority of the patients were in New York Heart Association (NYHA) functional class II at admission. Seven (16.3%) patients were in NYHA class I, 33 (76.7%) patients were in NYHA class II, 1 (2.3%) patient was in NYHA class III (the right atrial myxoma was penetrating the right ventricle and leading to functional tricuspid obstruction), and 2 (4.7%) patients were in NYHA class IV (due to large left atrial myxoma filling the atrium and causing severe left ventricle inflow obstruction).

The tumor was located in the left atrium in 37 (86.0%) patients, in the right atrium in 5 (11.6%) patients, and in both the right atrium and right ventricle in 1 (2.3%) patient. Among the tumors originating in the left atrium, the most common implantation site was the interatrial septum (73%).

Coronary angiography was performed in 27 (62.8%) patients. Severe coronary artery disease requiring concomitant coronary artery bypass grafting surgery was detected in 5 patients. Furthermore, six patients with left atrial myxoma had moderatesevere mitral stenosis with or without mitral insufficiency requiring concomitant mitral valve surgery, two patients with right atrial myxoma had moderate-severe tricuspid stenosis with or without tricuspid insufficiency requiring concomitant tricuspid valve surgery. One patient had undergone concomitant wedge resection of upper lobe of the right lung due to a large mass identified by computed tomography scan. Concomitant surgical procedures and surgical approaches are shown in Table 2.

**Table 2:** Surgical approaches and concomitant surgical procedures

	No. of patients (%)
**Surgical approaches**	
Biatrial approach	34 (79.1)
Right atrial transseptal approach	17 (39.5)
Biatriotomy	15 (34.9)
Superior transseptal approach	2 (4.7)
Uniatrial approach	8 (18.6)
Left atriotomy	3 (7)
Right atriotomy	5 (11.6)
Right atriotomy + right ventriculotomy	1 (2.3)
**Concomitant procedures**	
Coronary artery bypass grafting	5 (11.6)
Mitral valve replacement	4 (9.3)
Mitral valve repair	2 (4.7)
Tricuspid valve replacement	1 (2.3)
Tricuspid valve repair	1 (2.3)
Lung wedge resection	1 (2.3)
Atrial septal defect repair	29 (67.4)
Pericardial patch	19 (44.2)
Dacron patch	4 (9.3)
Directly sutured	6 (14)

The tumor was completely resected in all of the patients. The resections of myxomas were achieved through biatrial approach in 34 patients with left atrial myxoma (right atrial transseptal approach in 17 patients, biatriotomy in 15 patients, superior transseptal approach in 2 patients), uniatrial approach in 8 patients (right atriotomy in 5 patients with right atrial myxoma, and left atriotomy in 3 patients with left atrial myxoma), and right atriotomy with right ventriculotomy in 1 patient. Biatrial approach was preferred in 91.9% (n=34/37) of patients with left atrial myxoma. Interatrial septum was resected with tumor in 29 patients and it was repaired using a pericardial patch in 19 patients, a Dacron patch in 4 patients, and by being directly sutured in 6 patients. Mean cardiopulmonary bypass time was 93.8±39.5 (range: 38-206) minutes and mean aortic crossclamp time was 70.3±25.1 (range: 27-148) minutes. Mean tumor dimension at the largest diameter was 5.8±3.2 cm (range 0.8-10.4 cm).

Short and long-term outcomes of patients are summarized in Table 3. There was no intraoperative death. Only one (2.3%) patient died due to low cardiac output syndrome in the early postoperative period. The patient had severe left ventricular dysfunction (ejection fraction: 25%) and also underwent concomitant coronary artery bypass grafting. After operation, he was taken to the intensive care unit (ICU) with intraaortic balloon pump (IABP) and inotropic support. In ICU follow-up, despite receiving full-dose inotropic support with IABP, he did not respond to therapy and died on the 6^th^ postoperative day.

**Table 3:** Short and long-term outcomes of the patients.

	No. of patients (%)
**Short-term outcomes (in 30 postoperative days)**	
Mortality	1 (2.3)
Atrial fibrillation	7 (16.3)
Bleeding (requiring surgical revision)	1 (2.3)
Pneumonia	1 (2.3)
Transient left hemiparesis	1 (2.3)
Wound infection	__
Incomplete resection	__
**Long-term outcomes**	
Recurrence	__
Myxoma related mortality	__
Overall mortality	6 (14)

Ten (23.3%) patients had early complications after operation, including: atrial fibrillation (AF) in 7 (16.3%) patients, postoperative bleeding requiring surgical revision in one (2.3%) patient, pneumonia in one (2.3%) patient, and transient left hemiparesis in one (2.3%) patient. AF was present preoperatively in three of the 7 patients with postoperative AF. The other 4 patients with new-onset AF were succesfully converted to sinus rhythm with medical cardioversion. Of those, both one patient who had previous AF and two patients with new-onset AF had undergone concomitant valve surgery together with myxoma resection. The patient who was reexplored due to bleeding on the 2nd postoperative day experienced a full recovery. The patient with postoperative pneumonia recovered after 14 days of treatment with antibiotics. The other complication of transient left hemiparesis spontaneously regressed. Mean length of ICU stay was 2.6±1.8 (range: 1-8) days and mean length of hospital stay was 7.8±3.7 (range: 5-22) days.

Complete follow-up was available for 39 of the patients, but three patients were lost to follow-up. No recurrence was observed during long-term follow-up. Five patients died from causes other than myxoma in the long-term follow-up period. One patient, who had undergone wedge resection of upper lobe of the right lung for bronchoalveolar carcinoma together with myxoma resection at the time of operation, died due to lung cancer on the 5^th^ postoperative month. One patient died due to coronary artery disease on the 7^th^ postoperative year, one patient died due to cerebrovascular event on the 11^th^ postoperative year, and the causes of death of the other two patients were unknown. The mean follow-up time was 102.3±66.5 (range: 2-242) months. Among the 34 survivors with complete follow-up available, 25 (73.5%) patients were in NYHA class I and 9 (26.5%) patients in NYHA class II at their last follow-up visits.

**Fig. 1:**
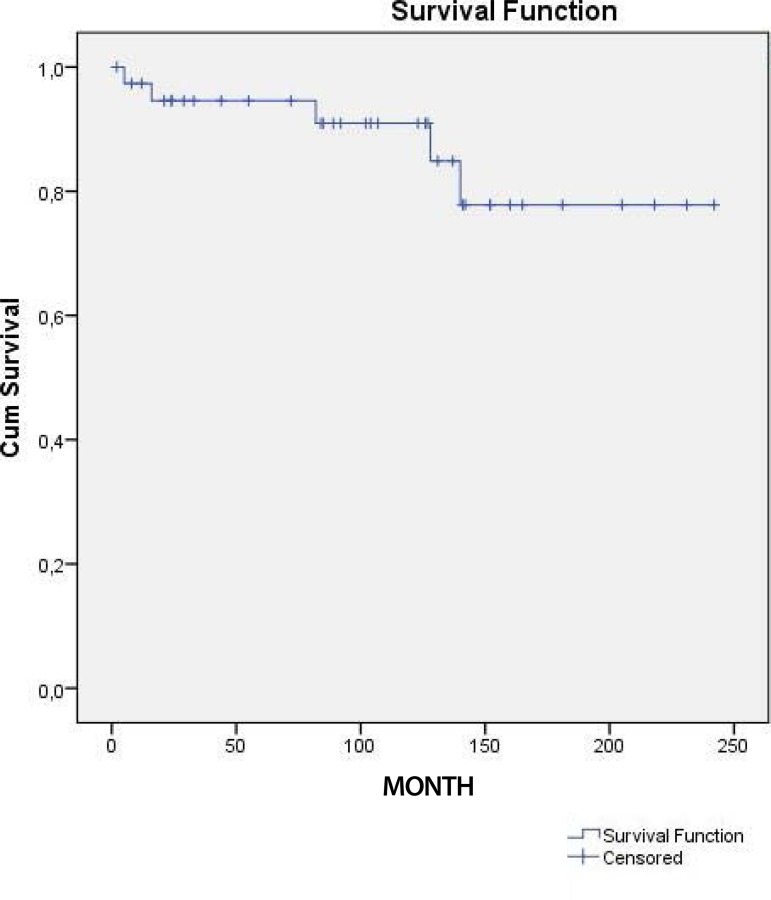
Kaplan-Meier cumulative survival curve of patients with cardiac myxoma who underwent surgical resection.

The actuarial survival rates were 95%, 92% and 78% at five, 10 and 15 years, respectively. Kaplan-Meier cumulative survival curve is shown in Figure 1.

## DISCUSSION

Primary cardiac tumors are often reported to have a low incidence and represent approximate 0.3% of all open heart surgeries^[13,14]^. Myxomas are the most common primary cardiac tumors. The ﬁrst successful resection of a cardiac tumor was a case with a left atrial myxoma reported by Crafoord, in 1954^[15]^. Nowadays, the surgical treatment of cardiac myxoma carries a low operative risk and is associated with excellent short and long-term outcomes in many cardiac surgery centers.

Myxomas are neoplasms of multipotent mesenchymal cells in the subendocardial tissue. The precise histogenetic origin has not been identified^[16]^. Histologically, the so-called myxoma cells are predominant. These polygonal, occasionally multinucleate, cells have an eosinophilic cytoplasm and are surrounded by a myxoid stroma. Degenerative changes such as cystic formations, hemorrhages, fibroses, and calcifications occur, as well as gland formation (lithomyxoma)^[17]^.

Cardiac myxomas usually occur in adults, most often between the third and sixth decades of life, and are predominantly present in females^[1,7-10]^. In our series, 23 (53.5%) patients were female, with a mean age of 51.7 years at the time of operation.

Myxomas may present with a wide spectrum of symptoms, from being completely asymptomatic to life-threatening catastrophic consequences. The structure, size and localization of the tumor are the most significant determinants of symptoms and outcomes. Most patients present with one or more of the triad of intracardiac obstruction, cerebral or peripheral embolisms, and constitutional symptoms and signs. Thus, the diagnosis usually requires physician suspicion. Incidental diagnosis during operation, autopsy studies, or routine echocardiographic examination for other purposes is not uncommon. In general, there are no specific physical examinations and/or laboratory findings related to myxomas. An auscultation finding, a "tumor plop" sound, which is an early diastolic murmur due to penetrating myxoma into the ventricle, is considered pathognomonic^[1,12,18]^. The symptoms may be intermittent, resulting from occasional prolapse of pedunculated and mobile tumors through the atrioventricular valves into the ventricle. Valvular obstruction may cause dyspnea, arrhythmias, precordial uneasiness, syncope, heart failure, acute pulmonary edema and sudden death. The level of atrioventricular valve obstruction depends on the site and size of the tumor. It may change depending on the position of the body. In patients with left atrial myxomas, the symptoms of left-sided heart failure, such as dyspnea on exertion, may progress to orthopnea, paroxysmal nocturnal dyspnea or pulmonary edema because of the obstruction at the mitral valve orifice. Systemic embolization may occur due to the friability of the tumor itself. The embolic symptoms are often nonspecific and minor embolic events such as transient visual loss and transient loss of consciousness are often overlooked. The most distressing is the embolization to the brain vessels, resulting in transient ischemic attack, reversible ischemic neurologic deficit or stroke, and persistent neurologic deficit both pre- and intraoperatively. The embolization is not related to the size of the myxoma and may occur with very small-sized tumors even before mechanical intervention occurs. The constitutional symptoms and signs are fever, fatigue, weakness, weight loss, anemia, arthralgia, and myalgia. Some investigations have revealed that it is associated with an immunologic basis, especially with high values of interleukin-6 in plasma^[19-21]^. In addition, unknown factors may also affect this phenomenon. In our series, the most common symptom was dyspnea, observed in 48.8% of the patients. The other common symptoms were palpitation (37.2%), chest pain (20.9%), and constitutional symptoms (34.9%). Additionally, 20.9% of the patients had a history of systemic embolization and 16.3% of the patients were asymptomatic.

Cardiac myxomas may be localized on any cardiac chamber or structure; however, in every publication in the literature, the most common site of myxoma has been stated as being in the left atrium, with the tumor base on the interatrial septum^[1-14]^. Myxomas may also arise from the right atrium, the ventricles, the atrioventricular valves, or may even originate from the atrial or ventricular free wall or appendages^[22-24]^. In our series, we observed the myxoma was located in the left atrium in 86% of the patients. In 12% and 2% of the patients, the myxoma was located in right atrium and in both right atrium and right ventricle, respectively.

Diagnosis of cardiac myxomas is usually possible by TTE, with a detection rate of 95.2%. TTE often defines the location, size, shape, attachment, and mobility of the tumor. In cases where there is doubt or image quality is not optimal, TEE may be the next choice of diagnostic tool^[25,26]^. Coronary angiography must be performed if coronary artery disease is suspected or the patient is older than 40 years old^[1,7,11]^. Computed tomographic scanning and cardiac magnetic resonance imaging produce high resolution images of the heart. Both can provide additional information regarding the extent of tumor within the heart or into adjacent extracardiac structures and may help in making further distinctions. Contrast enhancement can be used to differentiate tumors from thrombi since thrombi do not usually show enhancement and fat-suppression techniques may further define tumors such as lipomas^[27]^. Finally, these techniques provide well definition of lesions prior to any surgical intervention.

When a diagnosis of cardiac myxoma has been established, prompt surgical treatment should be performed without delay because of the high risk of sudden death from thromboembolism or valvular obstruction. The basic principles of surgical treatment for cardiac myxomas include complete resection of the tumor to avoid intraoperative embolization and the presence of residual tumor. Special care must be taken to avoid intraoperative embolization of the myxoma. Manipulation of the heart before the aortic cross-clamping must be minimized to avoid the risk of embolization. Adequate resection with negative clear margins is the cornerstone of tumor resection to avoid the risk of recurrence. The tumor must be removed with a 0.5-1 cm margin of tissue^[28]^. Sometimes extensive resections may be required and, in such cases, a remnant defect may be reconstructed with a patch. However, removal of the tumors at the vicinity of the conduction tissue or on the atrioventricular valves can be technically difficult and very risky. Limited resection confined to the subendocardial level, rather than big tissue removal, may be inevitable. In these patients, close follow-up is necessary to rule out possible recurrence. Furthermore, copious irrigation of all cardiac chambers must be applied to remove small tumor particles after resection of myxoma^[29-32]^. Therefore, it is important to choose the appropriate surgical approach to the myxomas. The surgical approach to the myxomas should allow minimal manipulation of the tumor, provide adequate exposure for its complete resection, enable inspection of all four cardiac chambers, minimize recurrence, and be safe and effective^[32,33]^.

The surgical approach to the myxomas may vary according to localization of the tumor. For resection of a right atrial myxoma, classical right atriotomy is the generally accepted approach. For resection of a left atrial myxoma, there are generally two approaches: uniatrial and biatrial. However, the ideal surgical approach to left atrial myxomas is still controversial and a consensus regarding the surgical approach has not been reached. We preferred the biatrial approach that includes biatriotomy, right atrial transseptal approach and superior transseptal approach for the left atrial myxomas in a large proportion of patients (92%) because it provides some advantages over the uniatrial approach.

The biatrial approach to the myxomas was popularized by Kabbani & Cooley, in 1973^[34]^. To prevent incomplete removal and recurrence, complete eradication of the base of implantation is necessary, and the biatrial approach offers an excellent visualization of the left and right cavities allowing easy manipulation of the tumor^[34]^. Jones et al.^[32]^ defined the advantages of the biatrial approach as being: i) definition of tumor pedicle by direct visualization, ii) minimal manipulation of the tumor, iii) adequate margins of excision, iv) inspection of all heart chambers, and v) secure closure of the atrial septal defect^[32]^. Although the biatrial approach offers an excellent exposure, it has been criticized as being responsible for a high incidence of arrhythmias and conduction disturbances after the resection of left atrial myxomas^[35,36]^. In our experience, no malignant arrhythmias were observed after surgery. Only AF occurred postoperatively as rhythm disturbance in 7 (16%) cases. AF was present preoperatively in three of the 7 cases with postoperative AF, and the other 4 cases with new-onset AF were succesfully converted to sinus rhythm medically. Furthermore, there was no correlation between the type of approach and the incidence of new-onset AF (one case with new-onset AF had uniatrial approach).

The biatrial approach was accepted as the classical approach; however, some studies which compare biatrial and uniatrial approaches have shown that an uniatrial incision is adequate to achieve similar outcomes^[31,37]^. Advocates of the uniatrial approach consider the exposure to be adequate and have demonstrated the low recurrence rates and safety with this approach. Interestingly, 34-85% had a subendocardial, not fullthickness resection of the interatrially based tumor. Nevertheless, there were no large patient series in those studies^[38,39]^. The uniatrial approach to left atrial myxomas is often inadequate, especially for large-sized tumors, because it requires excessive manipulation of the mass and, depending on the size of the tumor, may prevent adequate excisional margins to be obtained. Furthermore, this approach prevents inspection of all four cardiac chambers^[40]^. In our opinion, it is for those reasons that an uniatrial approach may fail to meet required surgical principles of left atrial myxoma resection. In our experience, we used the uniatrial approach to the left atrial myxomas in only 3 cases, with a small tumor arising from the left atrial posterior wall.

In a recent and intriguing study, Siminelakis et al.^[41]^ defined the ideal approach for the myxomas as being right atrial or both atrial incision with excision of the fossa ovalis and the surrounding tissues and closure with a pericardial patch, and the worst approach as the one through the left atrium, because the technique does not allow us to see properly the base and the petiole of the myxoma (there could be remaining tissue).

The minimal access surgery has become widespread in the milieu of cardiac surgery in last two decades. The minimally invasive video-assisted surgery for cardiac tumor resection is also becoming an exciting technique; recently, more cases with cardiac myxoma have received this novel procedure. Vistarini et al.^[42]^ and Schroeyers et al.^[43]^ have reported that minimally invasive video-assisted technique for myxoma resection is effective, safe, and a valuable alternative approach to standard sternotomy, with similar satisfactory outcomes. However, those reports have small case series and there are concerns in applying a minimally invasive approach to myxoma resection, because it may increase manipulation of tumor, thus raising the possibility of local and systemic embolization. Therefore, there is a requirement for larger case series and experiments.

Nowadays, the surgical treatment of cardiac myxomas may be successfully performed with low morbidity and mortality rates in many cardiac surgery centers. In several large case series, the early mortality rates have been 1-5%^[7,9-11,33,44,45]^. Our results were comparable with those reported in the literature; the early mortality rate was 2% in our series. In fact, early and late mortality could be related to the preoperative condition of the patients rather than cardiac or extracardiac conditions as well as to the age of the patient at the time of surgery.

Cardiac myxomas may recur postoperatively, but the mechanism of recurrent myxoma has not been clearly understood yet. Recurrence may occur within a few months to several years after the initial surgical excision and most recurrent myxomas are found during the first four years^[46]^. Atypical primary sites, insufficiency excision, metastasis, multicentricity and familial inheritance are all risk factors of the recurrent myxoma after surgery^[47]^. In the largest series in the literature, the recurrence rates of 2-6% have been reported^[11,44,45,48]^. In our study, we observed no recurrence in the 39 patients that completed the follow-up, with a mean follow-up time of 102 months. That is due to the fact that no familial or multiple myxomas were present in our series. However, Pacini et al.^[44]^ reported a recurrence rate of 4.4% in 91 myxoma survivors and all of recurrent myxoma cases were sporadic. We would like to emphasize that recurrence may be prevented with the appropriate surgical approach and technique. To prevent recurrence, complete tumor excision, including at least 5 mm normal area of clean tissue around it, is crucial. Hence, we recommend the opening of both atria because it provides adequate visualization and access for surgical resection of left atrial myxomas. Lastly, despite the very low incidence of recurrence, patients should be followed to evaluate tumor recurrence through annual echocardiography.

There were some limitations due to the retrospective nature of this study, especially considering the long-term follow-up. Over the years, advances in cardiac surgical techniques and myocardial protection strategies have improved patient survival and reduced postoperative morbidity. Echocardiographic techniques are also improved, providing highly detailed and accurate information during those years and allowing cardiologist and cardiac surgeons to better evaluate and follow up patients. Another limitation of this study was the lack of a control group, which precluded comparison of surgical approaches, because the number of patients was insufficient in the uniatrial approach group. Despite these limitations, we believe this study provides an opinion about the importance of surgical technique for preventing recurrence. Additionally, this study also provides insight into the natural history of cardiac myxomas, showing good long-term outcomes after surgical resection.

## CONCLUSION

Although cardiac myxomas are benign tumors, they should be treated surgically as soon as possible after diagnosis because of embolic complications and obstructive signs. To prevent recurrence, especially in cardiac myxomas which are located in the left atrium, preferred biatrial approach is suggested for complete resection of the tumor and to avoid residual tumor. Appropriate surgical technique gives nearly excellent results.

**Table:** 

Authors’ roles & responsibilities	
AY	Conception and study design; realization of operations and/or trials; analysis and/or data interpretation; manuscript writing or critical review of its content; final manuscript approval
DS	Conception and study design; realization of operations and/or trials; analysis and/or data interpretation; manuscript writing or critical review of its content; final manuscript approval
YV	Conception and study design; analysis and/or data interpretation; statistical analysis; manuscript writing or critical review of its content; final manuscript approval
SE	Conception and study design; realization of operations and/or trials; analysis and/or data interpretation; manuscript writing or critical review of its content; final manuscript approval
HÖ	Conception and study design; realization of operations and/or trials; analysis and/or data interpretation; manuscript writing or critical review of its content; final manuscript approval
